# Going to the South

**Published:** 1857-07

**Authors:** 


### HALL'S JOURNAL OF HEALTH.

OUR LEGITIMATE SCOPE IS ALMOST BOUNDLESS: FOR WHATEVER BEGETS PLEASURABLE AND HARMLESS FEELINGS, PROMOTES HEALTH; AND WHATEVER INDUCES DISAGREEABLE SENSATIONS, ENGENDERS DISEASE.

VOL. IV.] JULY, 1857. [NO. VII.

WE aim to show how disease may be avoided, and that it is best, when sickness comes, to take no medicine without consulting an educated physician.

### GOING TO THE SOUTH.

"MR. EDITOR:—Having spent the past winter in Florida for the relief of pulmonary weakness, I will venture to offer to your readers some of the results of my experience and observation, as bearing on the question, whether this oft-tried experiment is likely to prove successful in the cure or the alleviation of consumptive disease. Every invalid who goes to the South to spend the winter, has quite a sufficient appreciation of the advantages to be gained; but very few have any idea of the sacrifices involved, until they actually experience them. Strange enough is it, that consumptive persons should cling to the exploded notion that a warm climate simply, without any attendant aids, will effect their cure. Yet this seems to be the practical belief of almost every invalid who visits Florida. Few comparatively abandon this fallacy, till death stares them in the face. We do not exaggerate, when we say that the life led by the great proportion of invalids whom we saw during the past winter, was a life of moping, despondent indolence. The poor victims of consumption, for the most part, do nothing but eat and sleep, and brood over every sad symptom of their decaying bodies.

"But, under the most favorable circumstances possible, it is seriously to be questioned, whether a winter's residence in Florida is likely to benefit more than one consumptive person out of ten. Let us look at the daily round of the invalid's life. If he wants any of the comforts of civilization, he must take up his abode in a hotel or large boarding-house. There is no alternative to this, but to camp out in the woods. At breakfast in the morning, one finds himself in the company of sick people. Hollow cheeks, emaciated forms, are all around him. Ten chances to one, the conversation will be taken up with the symptoms of disease. I have sometimes almost wished myself deaf, when forced to listen through a whole meal, to a detailed account of some poor sufferer's maladies. One may be comparatively well; but companionship of this sort is enough almost to make him believe that he is sick.

"Breakfast done, some plan must be devised to occupy the forenoon. The best practicable thing, is a ride on horseback. You would like a companion, but think it better to go alone, than have the company of a sick man, to remind you at every step of your own infirmities. A lonely ride, then, through level pine barrens, is your only resort. There is no scenery to attract the eye, scarcely a trace of civilized life, nothing, absolutely nothing, to prevent your mind from brooding sadly over every real symptom of disease, and imagining a thousand that have no reality. *Such* exercise, submitted to *for the sake of itself*, with no ulterior object, is a poor prescription for the invalid.

"The day having worn itself tediously away, night is welcome, if one can forget his lonely exile and lose himself in quiet sleep. But often the stillness of the night is interrupted by the sound of some poor invalid's distressing cough, falling upon your wakeful ear, like an omen of the grave. Not even the night, shall release you from the dreadful consciousness that you are virtually imprisoned for long weary months in an hospital. Who, that has lived for a single month in such an atmosphere, cannot testify to its depressing influence? Add to all this, that almost every invalid in Florida is far away from all the comforts and endearments of home. No loved and familiar face is near to beguile the tedious hours of exile. How many have we known to turn their eyes wishfully back toward their distant domestic circle, and wish, but in vain, that they might only be spared to die amid its hallowed light. I must not weary the patience of your readers, though much more might be said, adverse to the experiment which so many consumptives are every winter trying, to little or no good purpose. For myself, I will say, in closing, that, after a winter's residence in Florida, under the most favorable circumstances, I have no inclination to repeat the experiment."

The above, from a professional gentleman of observation and culture, who had leisure and means to secure every possible advantage from a trip to the South, merits the mature consideration of every invalid who contemplates a journey thither, for the recovery of health. Nor should it pass unheeded by those, the multitude, whose ready advice is, "Go to the South," which to many involves not only the abandonment of their means of support, but an expenditure in addition, which is only to be met by large sacrifices and painful economies. We think the religious press, especially, would do a public good, by giving the entire communication a wide distribution.

